# Translational value of understanding brain–spinal interactions in persistent pain

**DOI:** 10.4103/NRR.NRR-D-25-00837

**Published:** 2025-10-30

**Authors:** Juhee Shin, Hyun Jun Jang, Boyoung Lee

**Affiliations:** Center for Cognition and Sociality, Institute for Basic Science, Daejeon, Republic of Korea

Neuropathic pain is a complex and debilitating condition caused by lesions or dysfunction within the somatosensory nervous system. Affecting an estimated 7%–10% of the global population, it presents with spontaneous pain, hyperalgesia, and allodynia, often accompanied by long-term emotional and cognitive consequences, such as depression and anxiety, which result in a reduced quality of life. Despite extensive research efforts, effective treatments remain limited. This limited efficacy likely stems, in part, from the heterogeneous nature of neuropathic pain, which varies widely across individuals in both clinical presentation and treatment responsiveness. To date, most preclinical studies have focused on localized changes in gene and protein expression, particularly within the spinal dorsal horn, offering only a partial view of the molecular processes sustaining pain.

However, pain encoding extends far beyond spinal mechanisms. It involves distributed neural processing across supraspinal structures such as the somatosensory cortex, thalamus, anterior cingulate cortex (ACC), and prefrontal cortex (PFC), regions that collectively shape the emotional and cognitive dimensions of pain (Steininger et al., 2025). These areas, along with descending modulatory systems, form the broader “pain matrix.” Despite their importance, supraspinal contributions to neuropathic pain remain underexplored. Recent studies, including hypothalamic deep brain stimulation, underscore the translational value of understanding brain–spinal interactions in persistent pain (Cho et al., 2024). Furthermore, observed functional asymmetries between ipsilateral and contralateral hemispheres, as well as dorsal–ventral specialization within the spinal cord, highlight the need for spatially nuanced investigations across the central nervous system (CNS).

Beyond neural circuits, molecular research has increasingly recognized glycosylation, particularly N-linked glycosylation, as a dynamic regulator of neuronal excitability, immune signaling, and synaptic architecture (Nowycky et al., 2014; Conroy et al., 2021; Klarić et al., 2023). Although glycosylation has been implicated in regional vulnerability in diseases such as Alzheimer’s disease, its spatial regulation in neuropathic pain remains largely unexplored. Aberrant glycan patterns have been reported in pain conditions such as diabetic neuropathy and chronic low back pain (Wu et al., 2023), suggesting a potential role in disease maintenance or progression. However, most findings to date are derived from plasma or homogenized tissue, limiting insight into region-specific regulation across the CNS.

To address this gap, we employed matrix-assisted laser desorption/ionization mass spectrometry imaging (MALDI MSI) to generate a spatially resolved glycomic atlas of the spinal cord and brain in a rat model of neuropathic pain. Our results revealed strikingly divergent and laterally asymmetric glycan signatures across pain-related circuits. These findings suggest that N-glycan expression patterns may constitute a hidden molecular code underlying persistent pain following spinal cord injury.

**Figure 1:**
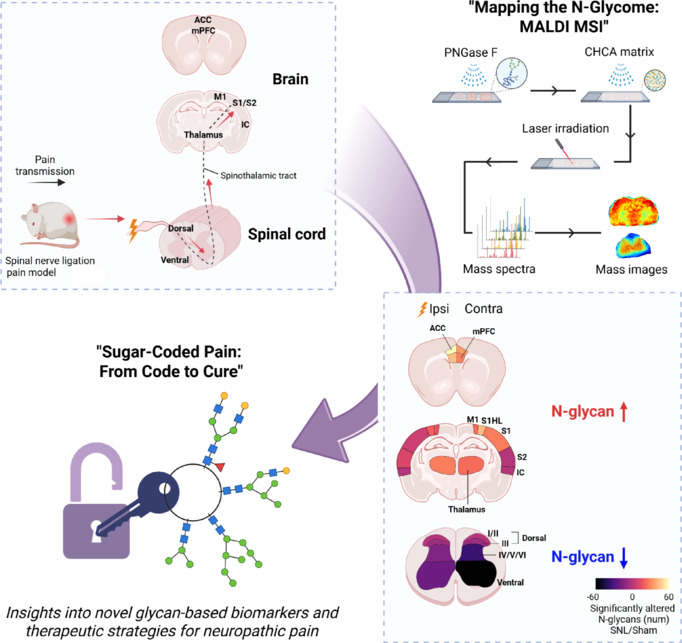
Dynamic alterations in N-glycosylation in the central nervous system following spinal nerve ligation. Bidirectional alterations in N-glycan expression in the spinal cord and brain during the post-acute phase following SNL may offer insights into novel glycan-based biomarkers and therapeutic strategies for neuropathic pain. The circle represents hexose, green represents mannose, and yellow represents galactose. Additionally, the blue square represents N-acetylglucosamine, and the red triangle represents fucose. Created with BioRender.com. ACC: Anterior cingulate cortex; CHCA: α-cyano-4-hydroxycinnamic acid; Contra: contralateral; IC: insular cortex; Ipsi: ipsilateral; M1: primary motor cortex; MALDI MSI: matrix-assisted laser desorption/ionization mass spectrometry imaging; mPFC: medial prefrontal cortex; PNGase F: peptide-N-glycosidase F; S1: primary somatosensory cortex; S1HL: hind limb region of the primary somatosensory cortex; S2: secondary somatosensory cortex.

**Dynamic alterations in N-glycosylation in the CNS following SNL:** Our recent study (Jang et al., 2025) is the first to comprehensively characterize N-glycan expression across both the spinal cord and multiple brain regions, many of which are core components of the pain matrix. Using MALDI MSI, we identified 65 distinct N-glycans in the spinal cord and 72 in the brain. At 14 days post-injury, a time point corresponding to the post-acute phase, we observed a predominant downregulation of N-glycans in the spinal cord of spinal nerve ligation (SNL) animals, in contrast to a widespread upregulation in multiple brain regions, compared to sham controls (**Figure 1**).

In the spinal cord, superficial layers (laminae I/II/III), which are typically associated with nociceptive relay, showed minimal N-glycan changes. In contrast, the deep dorsal horn (laminae IV/V/VI), involved in processing broader sensory input, exhibited the most significant reductions. Similarly, the ventral horn, responsible for motor output, showed substantial decreases. These findings suggest that areas involved in sensorimotor integration and compensatory motor control may undergo more dynamic N-glycosylation changes than those directly involved in nociception.

In the brain, regions linked to pain, including the thalamus, primary and secondary somatosensory cortices (S1, S2), S1HL (Hind limb region of the S1), primary motor cortex (M1), ACC, and medial PFC (mPFC), displayed significant N-glycan upregulation. These opposing patterns between spinal cord and brain point to region-specific regulatory mechanisms in response to neuropathic pain. Although structural and functional neuroplasticity in both spinal and supraspinal regions has been reported in neuropathic pain, the interpretation of bidirectional N-glycosylation changes remains unclear. While the functional consequences of these shifts have yet to be fully elucidated, our use of PNGase F, an enzyme that cleaves N-glycans from asparagine, suggests that these changes may reflect loss of glycoproteins or downregulation of glycosylation-related enzymes. To strengthen the functional relevance of region-specific N-glycan alterations, future studies should incorporate behavioral rescue experiments using region-specific glycosyltransferase inhibition (e.g., shRNA-mediated knockdown via viral delivery). In parallel, *ex vivo* electrophysiological recordings may elucidate whether glycan remodeling modulates neuronal excitability, thereby contributing to maladaptive neuroplasticity in neuropathic pain.

**Ipsilateral** ***versus*** **contralateral differences in N-glycan expression following SNL:** Pain signals originating ipsilaterally cross in the spinal cord and project contralaterally to the brain. We therefore examined whether lateralized N-glycosylation changes also occur. In the spinal cord, N-glycan alterations were more prominent on the contralateral side, particularly in laminae IV/V/VI and the ventral horn. Since rats tend to shift weight to the contralateral hindlimb to compensate for pain, these changes may reflect adaptive responses in sensorimotor circuits.

In the brain, although overall N-glycan levels did not show major ipsilateral–contralateral differences, the number of significantly altered glycans was notably higher in contralateral S1 and S1HL regions, critical for sensory processing. For instance, 6 glycans were altered in the ipsilateral S1 *versus* 24 in the contralateral side. Similar patterns were observed in S1HL. Volcano plot analysis revealed 17 significant glycan changes ipsilaterally and 36 contralaterally. These results suggest that even in the post-acute phase, dynamic glycosylation persists in brain regions involved in sensory processing.

Overall, our findings point to a potential role for N-glycan modulation in neuroplasticity related to pain. Since N-glycosylation contributes to protein stability, cell–cell communication, and synaptic function, further research should explore whether these changes are functionally linked to neural activity and the modulation of lateralized pain pathways.

**Implications for translating pain mechanisms into biomarkers:** The potential of region-specific N-glycosylation changes to serve as biomarkers for pain remains uncertain and requires further investigation. Although we observed dynamic alterations in many distinct N-glycan structures, it is difficult at this stage to pinpoint definitive biological markers. Moreover, since these changes were identified in tissue rather than in cerebrospinal fluid or blood, they may not directly serve as accessible biomarkers. Nevertheless, we investigated whether specific glycan structures exhibited consistent regional patterns. While overall changes in glycan composition were not uniformly distinct, certain brain regions showed significant alterations in particular glycan types. For instance, complex and hybrid fucosylated N-glycans were enriched in the ipsilateral dorsal laminae IV/V/VI, ACC, and both hemispheres of the mPFC, whereas changes in oligomannose structures were observed in the ipsilateral S1 and contralateral ACC (Supplementary Figure 17 from Jang et al., 2025). These findings suggest that region-specific glycosylation pathways may be active and that further identification of the underlying enzymatic regulators could yield novel therapeutic targets or biomarkers.

In addition, we identified one specific N-glycan structure, H6N6F2, composed of six hexoses, six N-acetylglucosamines, and two fucoses, that was significantly and consistently altered in three regions of the spinothalamic tract (Supplementary Figure 16 from Jang et al., 2025). Future studies could investigate whether peripheral glycan profiles from cerebrospinal fluid or serum reflect glycosylation signatures observed in central nervous system tissue, including H6N6F2. In this context, the development of glycan-specific positron emission tomography tracers, advanced imaging technologies, and enzyme activity modulators (such as fucosyltransferase inhibitors) holds promise for translating glycan biology into clinically relevant biomarkers and targeted therapeutic strategies for neuropathic pain.

**Challenges and future directions:** Our study reveals region-specific, dynamic N-glycosylation changes across the CNS in a neuropathic pain model. MALDI MSI enabled spatial visualization of glycosylation patterns in both spinal and supraspinal areas, emphasizing their relevance to sensory and emotional dimensions of pain. However, several challenges remain.

First, the mechanisms underlying divergent glycan patterns across CNS regions remain unclear. Following SNL, we observed opposing N-glycan expression patterns in the spinal cord and brain, suggesting that region-specific molecular changes may occur simultaneously in distinct areas of the central nervous system in response to pain. The underlying mechanisms remain unclear, and it is not yet known whether specific N-glycosylation pathways are directly involved in mediating this divergence. Although glycosylation studies in this context are limited, prior evidence supports the possibility of regionally distinct molecular responses. For example, in a CLN1 mouse model (Ppt1 deficiency), mitochondrial and synaptic proteins were decreased in the spinal cord but increased in the cortex (Nelvagal et al., 2020), possibly reflecting compensatory plasticity. Similarly, whether increased brain glycosylation following SNL reflects a compensatory response to spinal glycan loss remains to be determined.

Second, limited spatial resolution prevents cell type-specific interpretation of glycan changes. Understanding N-glycan alterations in the central nervous system is complicated by its high cellular diversity. Although our current analysis provides region-level profiling across spinal cord and brain, it does not clarify which specific cell types, such as microglia, astrocytes, or neurons, are responsible for the observed glycan changes. This limitation reflects the spatial resolution of MALDI MSI, which is not sufficient to distinguish glycan expression at the level of individual cell types. A promising direction involves the use of emerging single-cell glycomics, which is still in the early stages of development but holds strong potential to map glycan profiles with cellular precision. In parallel, established single-cell transcriptomic techniques can complement this approach by helping to identify glycosylation-related gene expression patterns across distinct cell populations. Applying these tools will be essential for determining the specific cellular contributors to regional glycan remodeling in neuropathic pain.

Third, the lack of sex-based analysis limits the translational relevance of our findings. Incorporating sex as a biological variable is another crucial direction. The current male-only design may impose limitations on the clinical translatability of our findings, particularly given known sex differences in pain prevalence and CNS activation patterns. Females often show greater pain sensitivity and distinct brain activation following nerve injury. For example, Murata et al. (2023) reported sex-dependent brain activity in macaques after peripheral nerve injury, with females showing heightened activation in the cingulate cortex, contralateral insular cortex, and somatosensory regions. Such findings suggest sex-specific central mechanisms in neuropathic pain, reinforcing the need to investigate sex-based glycosylation differences.

Fourth, spatial glycomics should be extended to the peripheral nervous system and multiple time points. Expanding glycosylation research beyond the CNS to the peripheral nervous system, especially the dorsal root ganglion, is equally important. The dorsal root ganglion plays a key role in pain signal relay and modulation, and its glycosylation profile may differ significantly from that of the CNS. Studying glycosylation in the dorsal root ganglion could reveal new therapeutic targets. Additionally, our study focused on tissue at a single time point—14 days post-SNL. Longitudinal studies tracking glycan changes at multiple stages could clarify the role of glycosylation in pain onset, progression, and persistence, and help identify optimal therapeutic windows.

Finally, O-glycosylation remains an understudied yet promising frontier in pain research. O-glycosylation, despite its relevance to neurological diseases (Prajitha et al., 2023), remains understudied in pain. A key barrier is the lack of enzymes that efficiently release intact O-glycans, hindering MSI-based approaches. Identifying such enzymes is critical for future progress. Meanwhile, alternative techniques such as liquid chromatography–mass spectrometry provide a viable route for investigating O-glycan changes, even in the absence of ideal enzymatic tools.

**Conclusion and final remarks:** Technological advances have propelled interest in neuroglycomics by allowing detailed analysis of brain glycans (Lee et al., 2024). Our findings show that neuropathic injury triggers dynamic, region-specific, and lateralized N-glycan changes in the CNS. The observed downregulation in the spinal cord versus upregulation in brain areas suggests a complex, topographically organized glycosylation landscape. These patterns challenge the view of glycosylation as merely a downstream process and support its active role in circuit-specific plasticity. By resolving glycosylation changes spatially, our study provides a molecular framework for understanding persistent pain.

These glycomic signatures, what we call a “sugar code,” may serve as biomarkers of pain states. Future work must uncover the molecular drivers of region- and glycan-specific alterations, identify key enzymes and glycoprotein targets, and map cell type-specific glycan profiles. Incorporating approaches such as peripheral glycan profiling (e.g., from cerebrospinal fluid or serum), along with single-cell glycomics and transcriptomics, may enhance biomarker discovery and help identify potential therapeutic targets in glycosylation-based pain research. Considering sex as a biological variable and extending research to peripheral structures like the dorsal root ganglion will also be critical. These efforts may pave the way for glycan-based diagnostics and therapies. Ultimately, a deeper understanding of spatial glycosylation dynamics could transform how we conceptualize and treat neuropathic pain through the lens of glycobiology.

*We thank Dr. Sangkyu Lee and Dr. Dong Woon Kim, the original authors of the study published in Jang et al., 2025, for their contributions, and Dr. C. Justin Lee from the Institute for Basic Science in Republic of Korea for his support and valuable comments on this perspective.*

*This work was financially supported by the Institute for Basic Science (IBS), Center for Cognition and Sociality (IBS-R001-D2 to BL).*
